# Assessment of Acute Kidney Injury and Longitudinal Kidney Function After Hospital Discharge Among Patients With and Without COVID-19

**DOI:** 10.1001/jamanetworkopen.2021.1095

**Published:** 2021-03-10

**Authors:** James Nugent, Abinet Aklilu, Yu Yamamoto, Michael Simonov, Fan Li, Aditya Biswas, Lama Ghazi, Jason Greenberg, Sherry Mansour, Dennis Moledina, F. Perry Wilson

**Affiliations:** 1 Section of Nephrology, Department of Pediatrics, Yale University School of Medicine, New Haven, Connecticut; 2 Clinical and Translational Research Accelerator, Department of Medicine, Yale University School of Medicine, New Haven, Connecticut; 3 Section of Nephrology, Department of Medicine, Yale University School of Medicine, New Haven, Connecticut; 4 Department of Biostatistics, Yale University School of Public Health, New Haven, Connecticut

## Abstract

**Importance:**

Acute kidney injury (AKI) occurs in up to half of patients hospitalized with coronavirus disease 2019 (COVID-19). The longitudinal effects of COVID-19–associated AKI on kidney function remain unknown.

**Objective:**

To compare the rate of change in estimated glomerular filtration rate (eGFR) after hospital discharge between patients with and without COVID-19 who experienced in-hospital AKI.

**Design, Setting, and Participants:**

A retrospective cohort study was conducted at 5 hospitals in Connecticut and Rhode Island from March 10 to August 31, 2020. Patients who were tested for COVID-19 and developed AKI were screened, and those who survived past discharge, did not require dialysis within 3 days of discharge, and had at least 1 outpatient creatinine level measurement following discharge were included.

**Exposures:**

Diagnosis of COVID-19.

**Main Outcomes and Measures:**

Mixed-effects models were used to assess the association between COVID-19–associated AKI and eGFR slope after discharge. The secondary outcome was the time to AKI recovery for the subgroup of patients whose kidney function had not returned to the baseline level by discharge.

**Results:**

A total of 182 patients with COVID-19–associated AKI and 1430 patients with AKI not associated with COVID-19 were included. The population included 813 women (50.4%); median age was 69.7 years (interquartile range, 58.9-78.9 years). Patients with COVID-19–associated AKI were more likely to be Black (73 [40.1%] vs 225 [15.7%]) or Hispanic (40 [22%] vs 126 [8.8%]) and had fewer comorbidities than those without COVID-19 but similar rates of preexisting chronic kidney disease and hypertension. Patients with COVID-19–associated AKI had a greater decrease in eGFR in the unadjusted model (−11.3; 95% CI, –22.1 to −0.4 mL/min/1.73 m^2^/y; *P* = .04) and after adjusting for baseline comorbidities (−12.4; 95% CI, –23.7 to −1.2 mL/min/1.73 m^2^/y; *P* = .03). In the fully adjusted model controlling for comorbidities, peak creatinine level, and in-hospital dialysis requirement, the eGFR slope difference persisted (−14.0; 95% CI, –25.1 to −2.9 mL/min/1.73 m^2^/y; *P* = .01). In the subgroup of patients who had not achieved AKI recovery by discharge (n = 319), COVID-19–associated AKI was associated with decreased kidney recovery during outpatient follow-up (adjusted hazard ratio, 0.57; 95% CI, 0.35-0.92).

**Conclusions and Relevance:**

In this cohort study of US patients who experienced in-hospital AKI, COVID-19–associated AKI was associated with a greater rate of eGFR decrease after discharge compared with AKI in patients without COVID-19, independent of underlying comorbidities or AKI severity. This eGFR trajectory may reinforce the importance of monitoring kidney function after AKI and studying interventions to limit kidney disease after COVID-19–associated AKI.

## Key Points

**Question:**

What is the association between coronavirus disease 2019 (COVID-19) in patients with acute kidney injury and the longitudinal trajectory of estimated glomerular filtration rate?

**Findings:**

In this cohort study of 1612 patients with acute kidney injury monitored after their index hospitalization, estimated glomerular filtration rate declined by 11.3 mL/min/1.73 m^2^ per year faster in patients with COVID-19–associated acute kidney injury compared with patients with acute kidney injury not associated with COVID-19. This finding persisted after adjusting for patient's baseline comorbidities and severity of acute kidney injury.

**Meaning:**

These findings suggest that patients recovering from COVID-19–associated acute kidney injury require monitoring of kidney function following hospital discharge.

## Teaser

This cohort study compares kidney function post discharge in patients with and without COVID-19 who experienced acute kidney injury during hospitalization.

## Introduction

Acute kidney injury (AKI) is common in patients hospitalized with coronavirus disease 2019 (COVID-19), reported in 24% to 57% of COVID-19 hospitalizations and 61% to 78% of intensive care unit admissions in patients with COVID-19.^1,2,3,4,5,6,7^ Compared with patients without COVID-19, those with COVID-19 develop more severe AKI, have greater dialysis requirements, and experience less in-hospital kidney recovery,^2^ which may increase their risk for incident chronic kidney disease (CKD) or progression of existing CKD.^8^

Although the acute effects of COVID-19 on kidney function have been studied,^9,10^ the intermediate- and long-term kidney outcomes after COVID-19–associated AKI remain unknown. Early follow-up of COVID-19 survivors with AKI has shown that 32% of patients had not yet recovered baseline kidney function at a median of 21 days after hospital discharge.^7^ Because the high incidence of COVID-19–associated AKI has strained health care delivery systems with limited dialysis resources,^11,12,13^ understanding the chronic kidney sequelae in this population has important public health implications for resource allocation, CKD screening, and patient counseling.^9^

The purpose of this retrospective cohort study was to describe the association between COVID-19 in patients with AKI and the rate of change in estimated glomerular filtration rate (eGFR) over the first 6 months after hospital discharge. In the absence of long-term follow-up data for patients with COVID-19–associated AKI, measuring the eGFR slope post hospitalization may inform prediction of future kidney disease progression.^14^ Owing to their more severe AKI, we hypothesized that patients with COVID-19–associated AKI are at increased risk for eGFR decrease or worsening CKD after discharge compared with patients with AKI who did not have COVID-19.

## Methods

### Population and Design

We included adults admitted and discharged at 5 hospitals within the Yale New Haven Health System network between March 10 and August 31, 2020, who received a reverse transcriptase–polymerase chain reaction test for severe acute respiratory syndrome coronavirus-2 (SARS-CoV-2). This study was approved by the Yale Human Investigation Committee, which waived the requirement of informed consent because the study relied on deidentified data from the electronic health record. This study followed the Strengthening the Reporting of Observational Studies in Epidemiology (STROBE) reporting guideline for cohort studies.^15^

Patients were included if they developed AKI during their hospitalization according to Kidney Disease: Improving Global Outcomes creatinine criteria, defined as either a 50% increase in the creatinine level over baseline or a 0.3-mg/dL (to convert to micromoles per liter, multiply by 88.4) increase from the lowest value within 48 hours.^16^ The baseline creatinine level was defined as either the lowest creatinine level within the previous 7 days or, when available, the median of all outpatient creatinine values obtained within 7 to 365 days before hospitalization. Acute kidney injury was classified into 3 stages according to Kidney Disease: Improving Global Outcomes creatinine criteria, with dialysis-requiring AKI classified as stage 3 regardless of the creatinine level. Urine output criteria were not used to define AKI owing to the high degree of missingness in this variable. To study the eGFR trajectory after hospital discharge, we included patients who survived past discharge, did not require dialysis within 3 days of discharge, and had at least 1 measurement of serum creatinine as an outpatient after discharge. We excluded patients who were younger than 18 years, had *International Statistical Classification of Diseases, 10th Revision* (*ICD-10*) codes for end-stage kidney disease, or kidney transplant (codes N18.6 or Z94) on a prior encounter, or had an initial hospital creatinine level greater than or equal to 4 mg/dL. For patients with multiple admissions during the study period, we only included data from their first admission. We did not collect data on patients who opted out of research participation (<1% of hospitalized patients).

Our exposure of interest was a diagnosis of COVID-19 by reverse transcriptase–polymerase chain reaction in patients who developed AKI during their hospitalization and survived past discharge. We compared patients with COVID-19–associated AKI with patients with AKI who tested negative for SARS-CoV-2 and were hospitalized during the study period. Testing for SARS-CoV-2 was performed at local or reference laboratories by nucleic acid detection methods, using oropharyngeal, nasopharyngeal, or a combination of oropharyngeal and nasopharyngeal swabs.

Our primary outcome was the rate of change in eGFR (ie, eGFR slope) from the time of discharge among patients with and without COVID-19–associated AKI who had at least 1 serum creatinine level measurement as outpatients following their hospitalization. The eGFR was calculated using the Chronic Kidney Disease Epidemiology Collaboration equation.^17^ The discharge serum creatinine level was defined as the last measured creatinine level before hospital discharge. We also evaluated the secondary outcome of AKI recovery, defined as a serum creatinine level less than 1.5 times the baseline creatinine level for the subgroup of patients who had not achieved AKI recovery by the time of discharge.

We extracted information on demographics, comorbidities (using *International Classification of Diseases, 9th Revision* and *ICD-10* codes), procedures, and medications from the electronic health record through the Yale Joint Data Analytics Team’s HELIX data repository. We validated key features, including dialysis, death, and the need for mechanical ventilation through manual medical records review of a random subsample. Race and ethnicity were extracted from the patient-reported demographic information in the electronic medical record, which is based on patients’ selections from a prespecified list of options. We included race and ethnicity as covariates because Black race has been independently associated with AKI^2,4,18^ and AKI severity^18^ among patients with COVID-19.

### Statistical Analysis

Descriptive characteristics for patients with and without COVID-19–associated AKI were compared with the χ^2^ test for proportions and Wilcoxon rank sum test for medians. To assess the association between COVID-19–associated AKI and eGFR slope after discharge, we constructed a linear mixed-effects model^14^ using random intercept and random slope terms with multiple lines per patient and tested the interaction between COVID-19 status and time since discharge. We present an unadjusted model, a model adjusted for baseline demographic characteristics (age, sex, race, body mass index, and hospital of admission) and comorbidities (congestive heart failure, hypertension, diabetes, baseline eGFR, and Elixhauser comorbidity score^19^), and a model adjusted for baseline characteristics and comorbidities, as well as peak serum creatinine level and whether dialysis was required during the index hospitalization. To evaluate for ascertainment bias in which patients at higher risk of eGFR decreases are more likely to have creatinine levels measured after discharge, we compared demographic and hospitalization-related risk factors for eGFR decreases between included patients and patients who were excluded owing to a lack of an outpatient creatinine measurement. To assess whether the results were sensitive to the fact that we included only patients with follow-up eGFR measures, we used an inverse propensity-weighted model,^20^ in which each patient was weighted according to the inverse probability of having at least 1 outpatient eGFR measurement given all baseline covariates. Because mixed-effects models can account for differential participant dropout based on previous observed eGFR values but not on the unobserved eGFR values,^21^ we tested a joint modeling approach to account for informative dropout.^22,23^ In an additional sensitivity analysis, we applied an alternative definition of AKI using a rolling baseline approach in which the lowest creatinine value within a 7-day rolling window before the current creatinine level was determined served as the baseline creatinine value to account for any differential missingness in prehospital outpatient baseline creatinine values. We also explored the association between in-hospital muscle loss and eGFR trajectory by running the mixed-effects model with weight loss added to the fully adjusted model. For the secondary outcome of AKI recovery, we used Cox proportional hazards regression to measure the association between COVID-19 status and time to AKI recovery. Survival functions between groups were compared using the log rank test. We performed complete case analysis because missingness in covariate data was less than 1%. With 2-tailed, unpaired testing, we defined statistical significance at *P* < .05. We conducted the statistical analysis using SAS, version 9.4 (SAS Institute Inc), Stata, release 15 (StataCorp LLC), and R, version 4.0.0 (R Project for Statistical Computing).

## Results

### Population Characteristics

**Figure 1.**
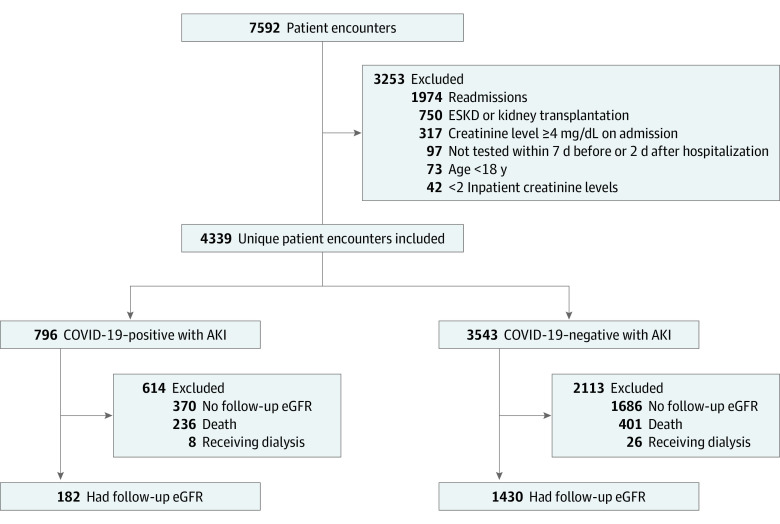
Study Flow Diagram AKI indicates acute kidney injury; COVID-19, coronavirus disease 2019; ESKD, end-stage kidney disease; and eGFR, estimated glomerular filtration rate.

The study population included 813 women (50.4%); median age was 69.7 years (interquartile range, 58.9-78.9 years). During the study period, 7592 encounters with AKI and a SARS-CoV-2 reverse transcriptase–polymerase chain reaction test occurred in a study hospital. Of the 4339 unique patient encounters eligible for further analysis, 182 patients with COVID-19–associated AKI and 1430 patients with AKI not associated with COVID-19 met the inclusion criteria (Figure 1). Patients with COVID-19–associated AKI were more likely to be Black (73 [40.1%] vs 225 [15.7%]) or Hispanic (40 [22%] vs 126 [8.8%]) and had fewer comorbidities than patients with AKI not related to COVID-19 but had similar rates of preexisting CKD and hypertension (Table 1). A higher proportion of patients with vs without COVID-19–associated AKI were excluded due to death during the index hospitalization (29.6% vs 11.3%); however, the proportions excluded due to dialysis requirement within 3 days of discharge (1.0% vs 0.7%) or lack of an outpatient creatinine measurement post hospitalization (46.5% vs 47.6%) were similar between groups. For the 34 patients excluded due to requiring dialysis at discharge, 2 of 8 patients with COVID-19–associated AKI and 6 of 26 patients with AKI not associated with COVID-19 remained on dialysis 6 months after discharge, and an additional 6 patients without COVID-19–associated AKI died within 6 months after discharge. Among patients with AKI who had received a SARS-CoV-2 test, patients who were excluded due to lack of an outpatient creatinine measurement had fewer comorbidities compared with included patients; however, AKI stage, dialysis requirement, intensive care unit admission, and AKI recovery were similar between groups (eTable 1 in the Supplement).

**Table 1.** Characteristics of Patients With Acute Kidney Injury According to COVID-19 Status

Characteristic	With COVID-19 (n = 182)	Without COVID-19 (n = 1430)	*P* value
Demographic			
Age, median (IQR), y	67.4 (58.3-80.1)	69.9 (59-78.7)	.72
Sex, No. (%)			
Female	86 (47.3)	727 (50.8)	.36
Male	96 (52.7)	703 (49.2)	
Race, No. (%)			
Black	73 (40.1)	225 (15.7)	<.001
White	74 (40.7)	1067 (74.6)	
Asian	5 (2.7)	20 (1.4)	
Other^a^	30 (16.5)	118 (8.3)	
Ethnicity, No. (%)			
Hispanic	40 (22)	126 (8.8)	<.001
Non-Hispanic	142 (78)	1304 (91.2)	
BMI, median (IQR)	28.1 (24.1-36.3)	28.4 (24.1-34.3)	.70
Comorbidities, No. (%)			
Congestive heart failure	75 (41.2)	709 (49.6)	.03
Chronic obstructive pulmonary disease	82 (45.1)	667 (46.6)	.69
Liver disease	30 (16.5)	359 (25.1)	.01
Chronic kidney disease	60 (33)	501 (35)	.58
Hypertension	162 (89)	1267 (88.6)	.87
Diabetes	116 (63.7)	722 (50.5)	<.001
Elixhauser comorbidity score, median (IQR)	9 (7-12)	10 (7-13)	.02
Baseline kidney function, median (IQR)			
Baseline creatinine, mg/dL	1.1 (0.9-1.4)	1.1 (0.8-1.4)	.78
Baseline eGFR, mL/min/1.73 m^2^	65.8 (46.3-85.9)	63.9 (43.3-86.5)	.22
Hospitalization factors			
Proteinuria on admission, No. (%)	61 (33.5)	270 (18.9)	<.001
BUN on admission, median (IQR), mg/dL	24 (16-35)	25 (17-39)	.26
Peak BUN, median (IQR), mg/dL	44 (28-59)	36 (25-53)	<.001
Peak creatinine, median (IQR), mg/dL	1.7 (1.3-2.4)	1.7 (1.3-2.4)	.58
AKI stage, No. (%)			
1	122 (67)	1093 (76.4)	.006
2	42 (23.1)	225 (15.7)	.01
3	18 (9.9)	112 (7.8)	.34
Dialysis requirement, No. (%)	7 (3.8)	17 (1.2)	.005
Duration of inpatient dialysis, median (IQR), d	16.8 (5.7-33.9)	4 (1.0-8.8)	.05
Length of hospital stay, median (IQR), d	14.1 (9.1-23.6)	6.9 (4.1-12.2)	<.001
AKI recovery at discharge, No. (%)	150 (82.4)	1143 (79.9)	.43
Duration of in-hospital AKI, median (IQR), d	1.3 (1.0-2.7)	1.1 (0.7-2.1)	.04
ICU admission, No. (%)	67 (36.8)	536 (37.5)	.86
Length of ICU stay, median (IQR), d	6.6 (3.5- 13.7)	3.6 (1.86.8)	<.001
Ventilator requirement, No. (%)	52 (28.6)	166 (11.6)	<.001
Vasopressor requirement, No. (%)	49 (26.9)	261 (18.3)	.005
New-onset congestive heart failure, No. (%)	60 (33)	607 (42.4)	.003
Loop diuretic administration, No. (%)	104 (57.1)	714 (49.9)	<.001
Systemic corticosteroid administration, No. (%)	72 (39.6)	304 (21.3)	<.001
Discharge creatinine, median (IQR), mg/dL	1 (0.7-1.4)	1.1 (0.8-1.6)	.003
Discharge eGFR, median (IQR), mL/min/1.73m^2^	68.5 (48.2-93.8)	58.4 (37.6-84.8)	<.001
Discharge BUN, median (IQR), mg/dL	20.5 (14-31)	22 (14-35)	.21
ACEi or ARB at discharge, No. (%)	43 (23.6)	291 (20.3)	.30
Weight change during admission, median (IQR), kg	–2.4 (–6.0 to 0.6)	–0.2 (–3.7 to 2.0)	<.001
Follow-up information			
Duration of follow-up, median (IQR), d	92.9 (52.5-127.7)	60.9 (30.0-102.9)	<.001
No. of serum creatinine measurements after discharge, median (IQR)	1 (1-3)	2 (1-4)	<.001
Postdischarge measurement, No. (%)			
1	96 (52.7)	554 (38.7)	<.001
2	34 (18.7)	255 (17.8)	.78
≥3	52 (28.6)	621 (43.4)	<.001
Death post-discharge, No. (%)	3 (1.65)	77 (5.38)	<.001

Abbreviations: ACEi, angiotensin-converting enzyme inhibitor; AKI, acute kidney injury; ARB, angiotensin receptor blocker; BMI, body mass index (calculated as weight in kilograms divided by height in meters squared); BUN, blood urea nitrogen; COVID-19, coronavirus disease 2019; eGFR, estimated glomerular filtration rate; ICU, intensive care unit; IQR, interquartile range.

SI conversion: To convert BUN to millimoles per liter, multiply by 0.357; creatinine to micromoles per liter, multiply by 88.4.

^a^ Race and ethnicity were extracted from the patient-reported demographic information in the electronic medical record, which is based on patients’ selections from a prespecified list of options. Other includes American Indian or Alaska Native, Native Hawaiian or Other Pacific Islander, Other Pacific Islander, Other/Not Listed, Patient Refused, and Unknown.

Baseline creatinine level and eGFR were similar between patients with and without COVID-19–associated AKI, while proteinuria on presentation was more common in patients with COVID-19–associated AKI. Patients with COVID-19–associated AKI were more likely to require dialysis, had longer hospital and intensive care unit stays, and had higher rates of mechanical ventilation and vasopressor use. Angiotensin-converting enzyme inhibitor or angiotensin receptor blocker prescriptions at discharge were similar between groups. Discharge eGFR was higher in patients with COVID-19–associated AKI. Patients with COVID-19–associated AKI had a longer duration of follow-up, defined as time from discharge to last outpatient creatinine measurement, while patients without COVID-19–associated AKI had more outpatient creatinine measurements after discharge.

### Primary Outcome

**Figure 2.**
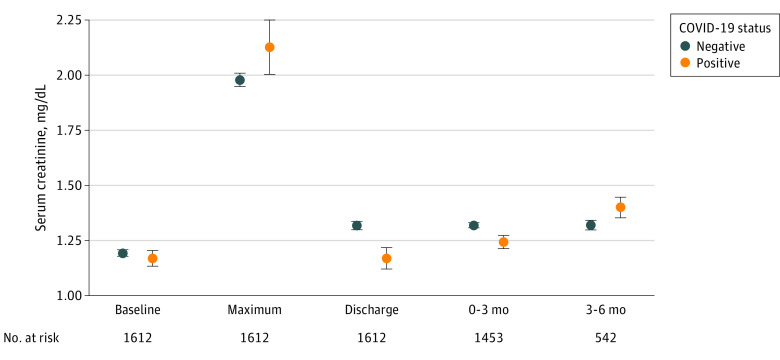
Measurement of Serum Creatinine at Different Time Points From Baseline to 6 Months After Hospital Discharge for Patients With Coronavirus Disease 2019 (COVID-19) Acute Kidney Injury (AKI) and Those With AKI Not Associated With COVID-19 Mean (SE) serum creatinine measurements are shown at different time points from baseline to 6 months after hospital discharge for patients with and without COVID-19–associated AKI. To convert serum creatinine to micromoles per liter, multiply by 88.4. Error bars indicate SE.

The mean serum creatinine level for each group at different time points during the observation period is displayed in Figure 2. In the unadjusted mixed-effects model, the mean rate of eGFR decline was –11.3 mL/min/1.73 m^2^/y (95% CI, –22.1 to –0.4 mL/min/1.73 m^2^/y) faster for patients with COVID-19–associated AKI (*P* = .04) (Table 2). The difference in eGFR slope persisted after adjusting for baseline demographic characteristics and comorbidities (–12.4; 95% CI, –23.7 to –1.2 mL/min/1.73 m^2^/y; *P* = .03). In the fully adjusted model including both baseline patient characteristics and comorbidities as well as peak serum creatinine levels and dialysis requirements, patients with COVID-19–associated AKI continued to show an increased rate of eGFR decrease (–14.0; 95% CI, –25.1 to –2.9 mL/min/1.73 m^2^/y; *P* = .01). In a sensitivity analysis, weighting the model by the inverse propensity of having a follow-up eGFR measurement did not substantially change the results (eTable 2 in the Supplement) (slope difference, –14.9; 95% CI, –26.7 to –3.1 mL/min/1.73 m^2^/y; *P* = .01). The results were also similar in the joint model (eTable 3 in the Supplement) (slope difference, –12.2; 95% CI, –19.8 to –4.2 mL/min/1.73 m^2^/y; *P* = .002), with a slightly narrower interval estimate owing to the additional information examined within the survival submodel for dropout. To account for differential missingness in outpatient baseline creatinine values (COVID-19–associated AKI, 40.5% vs AKI without COVID-19, 28.9%), we tested an alternative definition for AKI using only creatinine values measured during hospitalization as the baseline. The results were similar to the primary analysis (eTable 4 in the Supplement) (slope difference, –14.5; 95% CI, –27.5 to –1.5 mL/min/1.73 m^2^/y; *P* = .03). We explored in-hospital muscle loss by including weight loss in the fully adjusted model and the slope difference persisted (–15.3; 95% CI, –27.1 to –3.5 mL/min/1.73 m^2^/y; *P* = .01).

**Table 2.** Mean eGFR Slope After Hospital Discharge for Patients With vs Without COVID-19–Associated AKI

Variable	Unadjusted mean eGFR slope (95% CI)	*P* value	Adjusted mean eGFR slope^a^ (95% CI)	*P* value	Adjusted mean eGFR slope^b^ (95% CI)	*P* value
Difference in slope	–11.3 (–22.1 to –0.4)	.04	–12.4 (–23.7 to –1.2)	.03	–14.0 (–25.1 to –2.9)	.01
AKI with COVID-19	–12.1 (–22.2 to –2.0)		–15.0 (–42.0 to 12.0)		–16.7 (–43.4 to 10.0)	
AKI without COVID-19	–0.8 (–4.9 to 3.2)		–2.6 (–26.9 to 21.8)		–2.7 (–26.8 to 21.4)	

Abbreviations: AKI, acute kidney injury; COVID-19, coronavirus disease 2019; eGFR, estimated glomerular filtration rate (mL/min/1.73 m^2^/y).

^a^ Adjusted for age, sex, race, body mass index, hospital of admission, baseline eGFR, Elixhauser comorbidity score, congestive heart failure, hypertension, and diabetes.

^b^ Adjusted for factors above plus peak creatinine level and dialysis requirement.

### Secondary Outcome

By the time of hospital discharge, 82.4% of patients with and 79.9% of patients without COVID-19–associated AKI had achieved AKI recovery. In the subgroup of patients who had not recovered at discharge (n = 319), patients with COVID-19–associated AKI recovered slower than patients with AKI not associated with COVID-19 after discharge and COVID-19–associated AKI was independently associated with a lower rate of kidney recovery during outpatient follow-up (adjusted hazard ratio, 0.57; 95% CI, 0.35-0.92) (Table 3; eFigure 1 in the Supplement). The mean serum creatinine level for each subgroup at different time points is displayed in eFigure 2 in the Supplement.

**Table 3.** Kidney Recovery After Discharge According to COVID-19 Status

Variable	AKI recovery rate per 100 patient-days (95% CI)	*P* value	Unadjusted HR (95% CI) for AKI recovery	*P* value	Adjusted^a^ HR (95% CI) for AKI recovery	*P* value
AKI with COVID-19 (n = 32)	0.95 (0.62-1.46)	.02	0.58 (0.37-0.91)	.02	0.57 (0.35-0.92)	.02
AKI without COVID-19 (n = 287)	1.73 (1.51-2.0)		NA		NA	

Abbreviations: AKI, acute kidney injury; COVID-19, coronavirus disease 2019; HR, hazard ratio; NA, not applicable.

^a^ Adjusted for age, sex, race, body mass index, hospital of admission, baseline estimated glomerular filtration rate, Elixhauser comorbidity score, congestive heart failure, hypertension, diabetes, peak creatinine level, and dialysis requirement.

## Discussion

In this cohort of patients with AKI either with or without COVID-19 followed up after hospital discharge, patients with COVID-19–associated AKI experienced greater eGFR decreases independent of patient demographic characteristics, comorbidities, and severity of the AKI episode. In the subgroup of patients who had not yet recovered baseline kidney function at discharge, patients with COVID-19–associated AKI were less likely to achieve kidney recovery during outpatient follow-up.

There is a well-established association between AKI and risk of CKD, with CKD risk increasing in a graded manner depending on the severity of an AKI episode.^8^ Because patients with COVID-19 develop more severe AKI compared with those without COVID-19,^2^ patients with COVID-19–associated AKI may be expected to have a faster eGFR decrease after discharge, as we observed in our study population, independent of a patient’s underlying comorbidities. The persistence of this outcome after adjusting for AKI severity, represented by peak creatinine levels and the need for dialysis, suggests that the accelerated eGFR decrease may be mediated by other markers of AKI severity, additional hospitalization-related exposures associated with eGFR decrease, the hyperinflammatory state associated with COVID-19, or residual direct effects of SARS-CoV-2.

The pathogenic mechanism by which SARS-CoV-2 causes AKI is likely multifactorial. The most common histologic diagnosis noted on kidney biopsy in patients with COVID-19–associated AKI is acute tubular injury,^24,25^ which may reflect the multiple indirect causes of AKI associated with critical illness, including hemodynamic instability, acute respiratory distress syndrome, excessive diuresis, nephrotoxin exposures, hypoxia, cytokine storm, rhabdomyolysis, and secondary infections. In addition to these indirect effects, detection of SARS-CoV-2 RNA^26,27,28^ and live virus^26^ in the kidneys of patients with COVID-19 supports the hypothesis that SARS-CoV-2 may display a direct kidney tropism via angiotensin-converting enzyme-2 receptors expressed on proximal tubule cells and podocytes.^29^ The pathogenesis of continued kidney dysfunction in our cohort after recovery from COVID-19–associated AKI is unclear. The presence of lung fibrosis observed after pulmonary infections from other coronavirus strains^30^ has raised concern that COVID-19–associated AKI may induce tubulointerstitial fibrosis,^9^ which is a potential pathway for the progression of AKI to CKD.^31,32^

Although the primary finding of acute tubular injury on kidney biopsy may be suggestive of reversible injury with COVID-19–associated AKI,^24^ our results suggest that patients with COVID-19–associated AKI are at higher risk for acute kidney disease, defined as an ongoing subacute loss of kidney function between 7 and 90 days after an AKI-initiating event.^16,33^ Patients with AKI that evolves into acute kidney disease show greater long-term eGFR decreases than patients with AKI who do not develop acute kidney disease.^34^ Older age, male sex, Black race, underlying CKD, and AKI severity are known risk factors for AKI progression to acute kidney disease ^35,36^; however, the accelerated eGFR decrease following COVID-19–associated AKI persisted after adjusting for these covariates. As in acute tubular necrosis, where patients with acute tubular necrosis due to mixed causes are at higher risk for future CKD than patients with acute tubular necrosis due to a single cause, such as ischemia or nephrotoxins,^37^ the multiple concurrent mechanisms of COVID-19–associated AKI may be contributing to the increased eGFR decrease after discharge. Although the eGFR slope after AKI has not been widely described, the eGFR slope in our patients with AKI not associated with COVID-19 is similar to the eGFR slope seen in patients after AKI due to coronary angiography.^38^ Our study’s inclusion of a comparison group of patients with AKI not associated with COVID-19 followed up longitudinally allows for testing of the hypothesis that COVID-19–associated AKI displays not only unique clinical and pathologic features, but also distinct sequelae from other causes of AKI. Optimizing blood pressure control, reconciling medications to avoid nephrotoxins, and evaluating indications for renin-angiotensin-aldosterone system blockade may be opportunities to slow disease progression in early outpatient follow-up.^39^

### Limitations

One limitation of our study is that approximately 45% of patients with AKI in both groups were excluded because they did not have an outpatient measurement of their creatinine level after discharge. In addition, AKI stage, need for dialysis, and AKI recovery by discharge were similar between patients included in the final analysis and patients who were lost to follow-up. Although outpatient follow-up in our study population may have been impeded by the pandemic, incomplete follow-up after AKI has been seen in the prepandemic setting, with reports that 57% of patients with AKI requiring in-hospital dialysis have creatinine levels measured within 6 months of discharge^40^ and 12% to 18% have followed up with a nephrologist.^40,41^ The consistency of our results in the inverse propensity-weighted model and joint model suggests that the findings may still be generalizable to the population of patients who did not have close outpatient follow-up. The eGFR trajectory observed after COVID-19–associated AKI underscores the importance of quality improvement efforts^39,42^ and clinical practice guidelines^16^ recommending follow-up serum creatinine level measurement within 3 months of discharge after AKI.

Assessing kidney function using serum creatinine levels may be affected by changes in muscle mass, and the lower mean serum creatinine level at discharge in the COVID-19–associated AKI group may reflect greater muscle loss from their longer hospitalizations or other factors related to their COVID-19 illness. The persistent eGFR slope difference after adjusting for in-hospital weight loss suggests that regaining muscle after discharge does not explain the observed eGFR decrease in the COVID-19–associated AKI group. Although follow-up time varied between the 2 groups, the mixed-effects model has been shown to provide unbiased effect estimates with an unbalanced number of creatinine measurements.^21^ The similarity of results between the mixed-effects model and joint model also suggested little evidence on the differential follow-up time as a result of informative dropout and supported the validity of our analyses. However, the low number of postdischarge creatinine measurements combined with the smaller sample size of the COVID-19–associated AKI group may contribute to the large variance of the eGFR slope estimates. Furthermore, extrapolating the rate of future eGFR decline beyond this initial 6-month period may not be valid, as acute effects of the exposure may not persist for longer times or the eGFR may change in a nonlinear pattern over time.^43,44,45^ Nevertheless, the steep negative acute slope in the eGFR trajectory after COVID-19–associated AKI warrants confirmation in other cohorts and longer observation periods.

## Conclusions

In this cohort study of US patients with and without COVID-19 who experienced in-hospital AKI, patients with COVID-19–associated AKI demonstrated faster rates of eGFR decreases after hospital discharge, independent of a patient’s baseline comorbidities or AKI severity. Identifying predictors of longitudinal eGFR decrease in patients with COVID-19–associated AKI may help prioritize which patients need close outpatient follow-up during the pandemic. A better understanding of COVID-19–associated AKI should provide opportunities for clinical trials to improve outcomes and inform the guidelines of post-COVID-19–associated AKI outpatient management.

## Notes

Supplement. **eTable 1.** Characteristics of Included Patients and Patients Who Were Excluded Due to Lack of Serum Creatinine Measurement After Hospital Discharge **eTable 2.** Inverse Propensity-Weighted Model of eGFR Slope After Hospital Discharge for Patients with COVID-19 AKI and COVID-19 Negative AKI **eTable 3.** Joint Model of eGFR Slope After Hospital Discharge for Patients with COVID-19 AKI and COVID-19 Negative AKI **eTable 4.** Mean eGFR Slope After Hospital Discharge by COVID-19 Status Using Rolling Baseline Definition of AKI (N=1219) **eFigure 1.** Time to AKI Recovery After Hospital Discharge for Patients Who Had Not Returned to Baseline Kidney Function by Discharge **eFigure 2.** Measurement of Serum Creatinine at Different Time Points From Baseline to Six Months After Hospital Discharge for the Subgroup of Patients Who Had Not Returned to Baseline Kidney Function by Discharge
