# Missing Initials in Author Names

**DOI:** 10.1001/jamanetworkopen.2021.11225

**Published:** 2021-04-28

In the Original Investigation titled “Assessment of Acute Kidney Injury and Longitudinal Kidney Function After Hospital Discharge Among Patients With and Without COVID-19,”^1^ published March 10, 2021, middle initials were missing from 3 author names. The names should have been listed as Jason H. Greenberg, MD, MHS; Sherry G. Mansour, MD, MS; and Dennis G. Moledina, MD, PhD. This article has been corrected.^1^
