# Supplementary material for: Assessment of Acute Kidney Injury and Longitudinal Kidney Function After Hospital Discharge Among Patients With and Without COVID-19
Source: JAMA Netw Open. 2021 Mar 10;4(3):e211095. doi: 10.1001/jamanetworkopen.2021.1095 (PMC7948062; doi:10.1001/jamanetworkopen.2021.1095)
Supplement: Supplement. — eTable 1. Characteristics of Included Patients and Patients Who Were Excluded Due to Lack of Serum Creatinine Measurement After Hospital Discharge eTable 2. Inverse Propensity-Weighted Model of eGFR Slope After Hospital Discharge for Patients with COVID-19 AKI and COVID-19 Negative AKI eTable 3. Joint Model of eGFR Slope After Hospital Discharge for Patients with COVID-19 AKI and COVID-19 Negative AKI eTable 4. Mean eGFR Slope After Hospital Discharge by COVID-19 Status Using Rolling Baseline Definition of AKI (N=1219) eFigure 1. Time to AKI Recovery After Hospital Discharge for Patients Who Had Not Returned to Baseline Kidney Function by Discharge eFigure 2. Measurement of Serum Creatinine at Different Time Points From Baseline to Six Months After Hospital Discharge for the Subgroup of Patients Who Had Not Returned to Baseline Kidney Function by Discharge [file jamanetwopen-e211095-s001.pdf]

Supplementary Online Content

Nugent J, Aklilu A, Yamamoto Y, et al. Assessment of acute kidney injury and longitudinal kidney function after hospital discharge among patients with and without COVID-19. *JAMA Netw Open*. 2021;4(3):e211095. doi:10.1001/jamanetworkopen.2021.1095

eTable 1. Characteristics of Included Patients and Patients Who Were Excluded Due to Lack of Serum Creatinine Measurement After Hospital Discharge

eTable 2. Inverse Propensity-Weighted Model of eGFR Slope After Hospital Discharge for Patients with COVID-19 AKI and COVID-19 Negative AKI

eTable 3. Joint Model of eGFR Slope After Hospital Discharge for Patients with COVID-19 AKI and COVID-19 Negative AKI

eTable 4. Mean eGFR Slope After Hospital Discharge by COVID-19 Status Using Rolling Baseline Definition of AKI (N=1219)

eFigure 1. Time to AKI Recovery After Hospital Discharge for Patients Who Had Not Returned to Baseline Kidney Function by Discharge

eFigure 2. Measurement of Serum Creatinine at Different Time Points From Baseline to Six Months After Hospital Discharge for the Subgroup of Patients Who Had Not Returned to Baseline Kidney Function by Discharge

This supplementary material has been provided by the authors to give readers additional information about their work.

© 2021 Nugent J et al. *JAMA Network Open.*

eTable 1. Characteristics of Included Patients and Patients who were Excluded due to Lack of Serum Creatinine Measurement after Hospital Discharge

Variables	Included Patients (N=1612)	Excluded Patients (N=2056)	p-value
**Demographics**			
Age, years	69.7 (58.9,78.9)	69.3 (56.3,80.9)	0.63
Sex, Female	813 (50.4%)	1007 (49%)	0.38
Race, Black	298 (18.5%)	409 (19.9%)	0.28
Ethnicity, Hispanic	166 (10.3%)	294 (14.3%)	<0.001
Body mass index, kg/m^2	28.3 (24.1,34.5)	28.1 (23.7,34.1)	0.07
**Comorbidities**			
Congestive heart failure	784 (48.6%)	806 (39.2%)	<0.001
Chronic obstructive pulmonary disease	749 (46.5%)	855 (41.6%)	0.003
Liver disease	389 (24.1%)	419 (20.4%)	0.007
Chronic kidney disease	561 (34.8%)	531 (25.8%)	<0.001
Hypertension	1429 (88.6%)	1706 (83%)	<0.001
Diabetes mellitus	838 (52%)	985 (47.9%)	0.01
Elixhauser comorbidity score	9 (5,12)	7 (4,11)	<0.001
**Baseline Kidney Function**			
Baseline creatinine, mg/dL	1.1 (0.8,1.4)	1 (0.8,1.4)	0.02
Baseline eGFR, mL/min/1.73m^2	64.3 (43.6,86.4)	66.2 (43.8,91.3)	0.01
**Hospitalization Factors**			
Proteinuria on admission	331 (20.5%)	411 (20%)	0.61
Peak creatinine, mg/dL	1.7 (1.3,2.4)	1.6 (1.2,2.2)	<0.001
Stage 1 AKI	1215 (75.4%)	1591 (77.4%)	0.15
Stage 2 AKI	267 (16.6%)	326 (15.9%)	0.56
Stage 3 AKI	130 (8.1%)	139 (6.8%)	0.13
Dialysis requirement	24 (1.5%)	34 (1.7%)	0.69
Duration of inpatient dialysis, days	7.1 (1.1,15.7)	7 (1.5,22.6)	0.65
Length of hospital stay, days	7.6 (4.3,13.5)	8 (4.5,15.5)	0.004
AKI recovery at discharge	1293 (80.2%)	1640 (79.8%)	0.74
Duration of in-hospital AKI, days	1.2 (0.8,2.2)	1.1 (0.8,2.1)	0.14
ICU admission	603 (37.4%)	802 (39%)	0.32
Length of ICU stay, days	3.8 (1.9,7.3)	3.9 (1.8,9.6)	0.15
Ventilator requirement	218 (13.5%)	338 (16.4%)	0.01
Vasopressor requirement	310 (19.2%)	421 (20.5%)	0.35
Discharge creatinine, mg/dL	1.1 (0.8,1.6)	1 (0.8,1.4)	<0.001
Discharge eGFR, mL/min/1.73m^2	59.5 (38.2,86.1)	68.3 (45.4,93.8)	<0.001
Delta creatinine (discharge – baseline), mg/dL	0 (-0.2,0.3)	0 (-0.2,0.2)	<0.001
ACEi or ARB at discharge	334 (20.7%)	407 (19.8%)	0.50
Loop diuretic administration	818 (50.7%)	873 (42.5%)	<0.001
Systemic corticosteroid administration	376 (23.3%)	450 (21.9%)	0.30

eGFR, estimated glomerular filtration rate; AKI, acute kidney injury, ICU intensive care unit; ACEi, ACE-inhibitor; ARB, angiotensin receptor blocker
Data expressed as median (IQR) or No. (%).

© 2021 Nugent J et al. *JAMA Network Open.*

eTable 2. Inverse Propensity-Weighted Model of eGFR Slope After Hospital Discharge for Patients with COVID-19 AKI and COVID-19 negative AKI

	Unadjusted mean eGFR slope (95% CI)	p-value	Adjusted mean eGFR slope * (95% CI)	p-value	Adjusted mean eGFR slope ** (95% CI)	p-value
Difference in Slope	-12.1 (-23.8, -0.4)	0.04	-13.3 (-25.2, -1.3)	0.03	-14.9 (-26.7, -3.1)	0.01
COVID+ AKI	-12.1 (-23.0, -1.1)		-17.4 (-45.5, 10.8)		-17.2 (-45.0, 10.6)	
COVID- AKI	0.05 (-4.1, 4.2)		-4.1 (-29.4, 21.2)		-2.3 (-27.3, 22.6)	

eGFR, estimated glomerular filtration rate (mL/min/1.73m^2/yr); AKI, acute kidney injury; CI, confidence interval
** adjusted for age, sex, race, body mass index, hospital of admission, baseline eGFR, Elixhauser comorbidity score, congestive heart failure, hypertension, diabetes mellitus*
*** adjusted for * plus peak creatinine and dialysis requirement*

© 2021 Nugent J et al. *JAMA Network Open.*

eTable 3. Joint Model of eGFR Slope After Hospital Discharge for Patients with COVID AKI and COVID-negative AKI						
	**Unadjusted mean eGFR slope (95% Bayesian Credible Interval)**	**p-value**	**Adjusted mean eGFR slope * (95% Bayesian Credible Interval)**	**p-value**	**Adjusted mean eGFR slope ** (95% Bayesian Credible Interval)**	**p-value**
Difference in Slope	-10.0 (-17.8, -2.3)	0.009	-10.9 (-19.1, -3.0)	0.007	-12.2 (-19.8, -4.2)	0.002
COVID+ AKI	-11.0 (-18.3, -3.7)		-10.0 (-23.4, 3.3)		-11.1 (-24.3, 2.4)	
COVID- AKI	-1.0 (-3.7, 1.9)		0.9 (-10.0, 11.3)		1.1 (-9.4, 12.1)	
Association parameter	-0.005 (-0.01, 0.003)	0.24	-0.005 (-0.01, 0.003)	0.39	-0.01 (-0.03, 0.01)	0.26

eGFR, estimated glomerular filtration rate (mL/min/1.73m^2/yr); AKI, acute kidney injury; CI, confidence interval

** Both submodels in the joint model (linear mixed model and survival mixed model) were adjusted for age, sex, race, body mass index, hospital of admission, baseline eGFR, Elixhauser comorbidity score, congestive heart failure, hypertension, diabetes mellitus*

*** Both submodels were adjusted for * plus peak creatinine and dialysis requirement*

© 2021 Nugent J et al. *JAMA Network Open.*

eTable 4. Mean eGFR Slope After Hospital Discharge by COVID-19 Status Using Rolling Baseline Definition of AKI (N=1219)[1]

	Unadjusted mean eGFR slope (95% CI)	p-value	Adjusted mean eGFR slope * (95% CI)	p-value	Adjusted mean eGFR slope ** (95% CI)	p-value
Difference in Slope	-10.8 (-23.5, 1.9)	0.09	-12.9 (-26.2, 0.3)	0.06	-14.5 (-27.5, -1.5)	0.03
COVID+ AKI	-9.4 (-21.2, 2.3)		-24.7 (-56.5, 7.2)		-24.4(-55.7, 6.9)	
COVID- AKI	1.4 (-3.4, 6.3)		11.7 (-40.5, 17.0)		-9.9 (-38.1, 18.3)	

eGFR, estimated glomerular filtration rate (mL/min/1.73m^2/yr); AKI, acute kidney injury; CI, confidence interval

[1]A total of 34 COVID+ patients and 359 COVID- patients were excluded from this sensitivity analysis due to meeting AKI criteria based on their prehospital outpatient baseline creatinine but not based on the rolling baseline approach.

** adjusted for age, sex, race, body mass index, hospital of admission, baseline eGFR, Elixhauser comorbidity score, congestive heart failure, hypertension, diabetes mellitus*

*** adjusted for * plus peak creatinine and dialysis requirement*

© 2021 Nugent J et al. *JAMA Network Open.*

eFigure 1. Time to AKI Recovery after Hospital Discharge for Patients who had not Returned to Baseline Kidney Function by Discharge

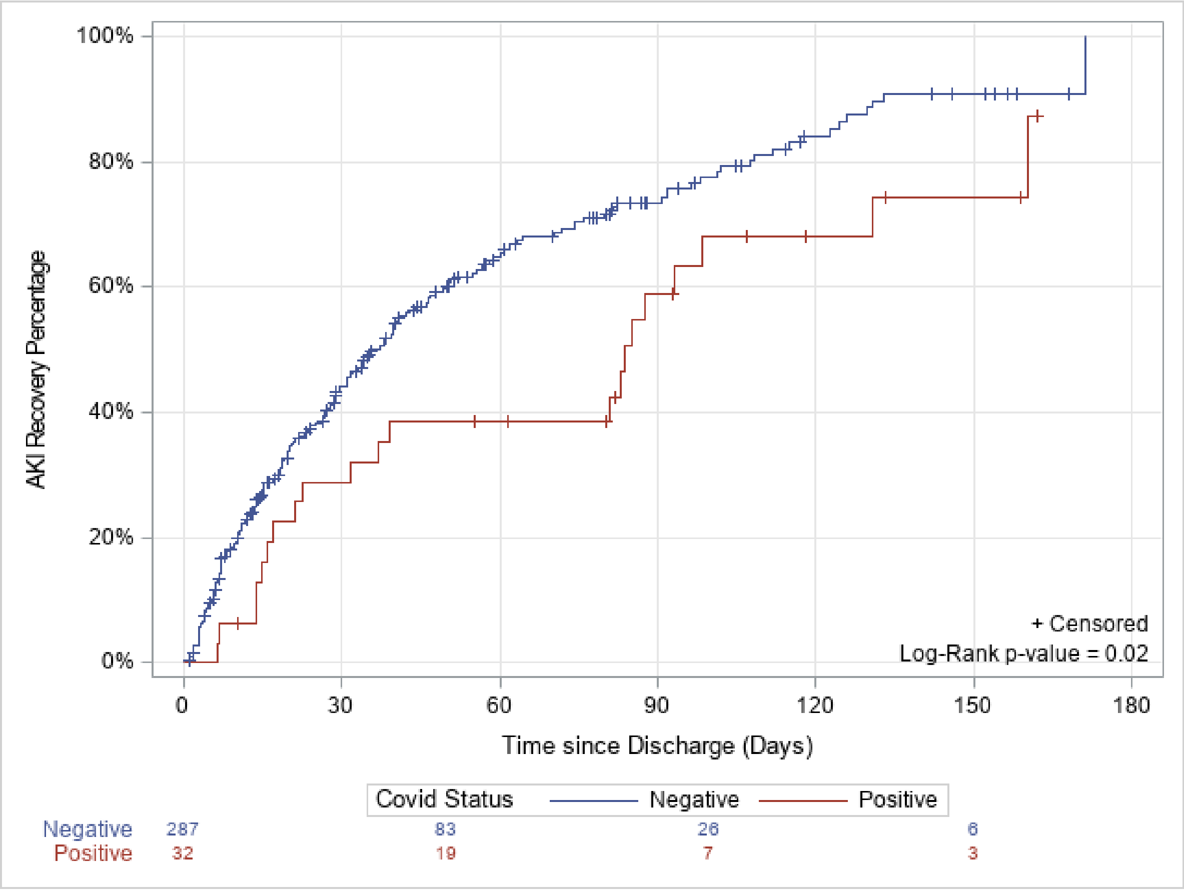

Survival curves for time to AKI recovery after discharge, defined as a serum creatinine <1.5 times the baseline creatinine, are shown for the subgroup of patients who had not achieved AKI recovery by the time of discharge from the hospital (n=319).
AKI, acute kidney injury

© 2021 Nugent J et al. *JAMA Network Open.*

eFigure 2. Measurement of Serum Creatinine at Different Time Points from Baseline to Six Months after Hospital Discharge for the Subgroup of Patients who had not Returned to Baseline Kidney Function by Discharge

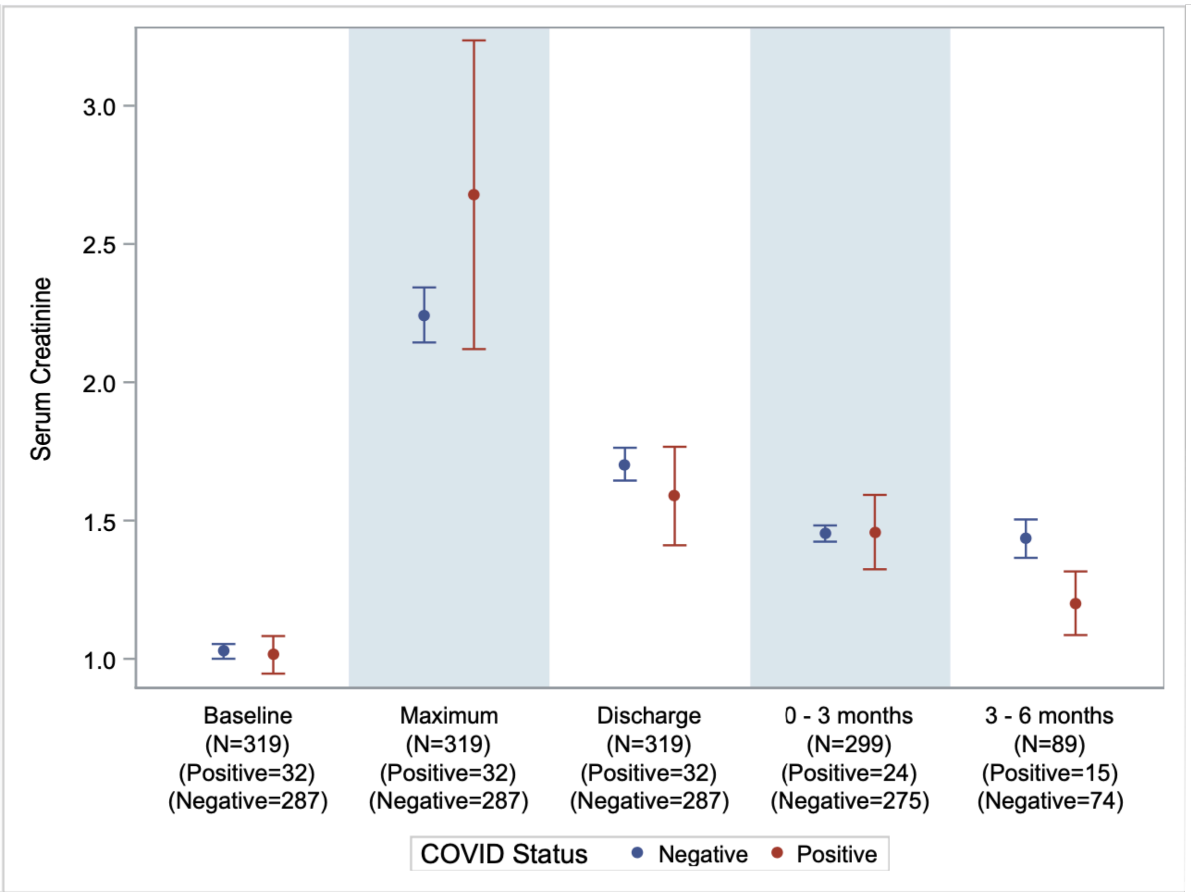

Mean (SE) serum creatinine measurements are shown at different time points from baseline to six months after hospital discharge for the subgroup of patients who had not returned to baseline kidney function by discharge.
AKI, acute kidney injury; SE, standard error

© 2021 Nugent J et al. *JAMA Network Open.*
